# Association between average glucose levels and hospital mortality among critically ill patients

**DOI:** 10.1186/cc12396

**Published:** 2013-03-19

**Authors:** B Yegneswaran, R Parket, S Gawel, A Pritchard-Bell, T Ho, G Clermont

**Affiliations:** 1 UPMC Hamot, Erie, PA, USA; 2 University of Pittsburgh, PA, USA

## Introduction

The objective of the study was to determine the association between average glucose level (AVG) and hospital outcomes in critically ill patients.

## Methods

We performed a chart review of the MIMICII and HIDENIC databases, prospective cohorts of over 32,000 and 46,000 patients admitted to the ICUs at Beth Israel Deaconess Medical Center between 2001 and 2007 and the ICUs at University of Pittsburgh Medical Center between 1991 and 2008, respectively. All admissions of patients ≥18 years without the diagnosis of DKA and NKHS were included. Diabetics were identified from ICD-9 documentation. Propensity score for death (pDead) was computed from either SAP1 (MIMICII) or APACHE III (HIDENIC) to assess the risk of death. Hypoglycemia was defined as AVG ≤60 mg/dl. AVG was computed as the area under the glucose curve throughout ICU admission. Mortality was examined within bins (each bin is categorized by a 10 mg/dl increase in AVG) and was compared between adjacent categories using a chi-square test. The same method was repeated among diabetics, nondiabetics, patients with lower (pDead greater than median) and higher (pDead lower than median) risk of death. Statistical significance was noted following a Bonferroni correction.

## Results

**Figure 1:**
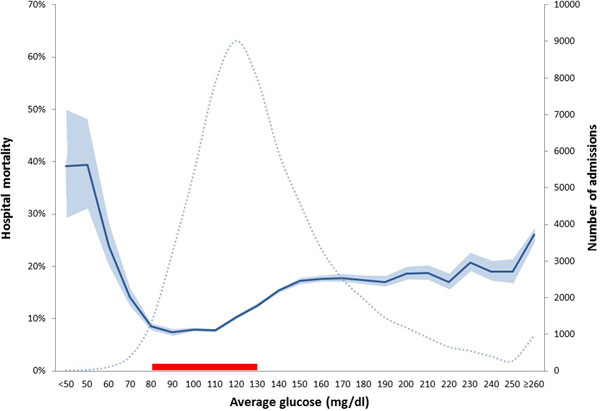


MIMICII database: 21,209 admissions met inclusion criteria. Mean age: 62.5 ± 16.4 years; female: 9,070 (42.8%); diabetes: 5,496 (25.9%); mean admission SAP1 score: 13.8 ± 5.2; mean LOS: 9 days (IQR 6.15); hospital mortality: 10.3%. HIDENIC database: 38,872 admissions met inclusion criteria. Mean age: 59.4 ± 17.3 years; female: 16,675 (42.9%); diabetes: 11,326 (29.1%); mean admission APACHE III score: 80.2 ± 14; mean LOS: 11 days (IQR 7.22); hospital mortality: 14.3%. In both databases, association between average glucose and hospital mortality followed a bathtub shape with mortality nadir between 80 and 130 mg/dl. No mortality difference was observed between adjacent AVG glucose bins in the bottom of the bathtub (Figure 1). This relationship was preserved in nondiabetics, and for patients with higher risk of death.

## Conclusion

Low and high average glucose are associated with mortality.

